# Status of Neutrophils, Lymphocytes and Platelets in Patients with Recurrent Aphthous Stomatitis: A Retrospective Study

**Published:** 2016-11

**Authors:** Suat Terzi, Engin Dursun, Abdulkadir Özgür, Enes Yiğit, Zerrin Özergin-Coşkun, Özlem Çelebi-Erdivanl, Münir Demirci, Metin Çeliker

**Affiliations:** 1 *Department of Otorhinolaryngology, Medical Faculty, Recep Tayyip Erdogan University, Rize, Turkey.*

**Keywords:** Blood cells, Neutrophil, Lymphocyte, Platelet, Recurrent aphthous stomatitis

## Abstract

**Introduction:**

The aim of this study was to evaluate the neutrophil-to-lymphocyte ratio (NLR), platelet-to-lymphocyte ratio (PLR), and mean platelet volume (MPV) in patients with recurrent aphthous stomatitis (RAS).

**Materials and Methods:**

Eighty patients who were diagnosed with RAS between January 2014 and January 2016 were included in this study. Eighty age- and gender-matched healthy subjects were also enrolled as a control group. Neutrophil, lymphocyte, and platelet counts were compared between groups, in addition to NLR, PLR, and MPV values.

**Results:**

There was no significant difference in terms of lymphocyte count, platelet count, PLR, or MPV values between the two groups (P>0.05). However, white blood count, neutrophil count, and NLR were significantly higher in patients with RAS compared with the control group (P<0.05).

**Conclusion:**

The present study revealed an increased NLR among RAS patients compared with healthy controls. This suggests that development of RAS involves an inflammatory process. We believe that NLR could be used as a cheap and simple marker of inflammation.

## Introduction

Recurrent aphthous stomatitis (RAS) is a common oral mucosal disease characterized by painful, well-demarcated, round or ovoid, single or multiple necrotizing ulcerations. It is among the most common oral mucosal lesions and affects about 10–20% of the population (1). Although several factors including genetics, nutritional, hormonal, allergic, psychological, traumatic, or infectious factors or inflammatory response are suspected in the etiology of RAS, none have been confirmed as contributing factors. RAS is currently believed to be idiopathic or to occur as a result of several trigger factors (2–4).

It is known that neutrophils and lymphocytes are responsible for inflammation. Neutrophil-to-lymphocyte ratio (NLR) and platelet-to-lymphocyte ratio (PLR) are novel biomarkers of subclinical inflammation and their use in the diagnosis and prognosis of various diseases has enjoyed increased popularity as an easy and practical method (5–8). Mean platelet volume (MPV) is another marker that reflects platelet activity and has been shown to be associated with inflammation and inflammation severity (8,9).

According to our literature research, there are only two studies evaluating the NLR and MPV ratio separately in patients with RAS. No such study has been conducted evaluating NLR, PLR, and MPV together in RAS. Therefore, we aimed to compare the values of NLR, PLR, and MPV in patients with RAS versus healthy controls.

## Materials and Methods

The study was conducted retrospectively. The archival records of patients who were admitted to the Department of Otorhinolaryngology of the Recep Tayyip Erdogan University Medicine Faculty, Rize, Turkey between January 2014 and January 2016 and diagnosed with RAS were reviewed. A diagnosis of RAS was based upon medical history and clinical findings. Demographic and laboratory data were extracted from the hospital management system. Patient data were reviewed retrospectively with respect to demographics, concomitant systemic diseases, and drugs used.

Eighty patients with RAS that recurred at least three times a year and that had no systemic manifestation were included in the study. Patients with Behçet ´s disease, a history of trauma, any systemic disease, or a history of smoking or alcohol abuse were excluded from the study. Eighty age- and gender-matched healthy subjects who came to our hospital for a regular health checkup and showed no signs of any systemic disease were also included in the study as the control group. Demographic characteristics and results of a complete blood count (CBC) were recorded for both groups. Leucocyte, neutrophil, lymphocyte, and MPV values were recorded from the CBC. NLR, PLR, and MPV were calculated as follows: NLR= neutrophil count/lymphocyte count and PLR= platelet count/lymphocyte count. The study was approved by the local ethics committee of the İstanbul Training and Research Hospital (820-08.04.2016) and was performed according to the principles of the Declaration of Helsinki. Written consent was obtained from all participants before hospitalization.

Data were analyzed using SPSS version 15.0 for Windows (SPSS Inc., Chicago, IL, USA). A Kolmogorov-Smirnov test was performed to determine whether the data followed a normal distribution. During the comparison of two independent groups, the Student t-test was used when the data were compatible with normal distribution. Otherwise, groups were compared by performing the Mann–Whitney U-test. A p-value less than 0.05 was considered to demonstrate significant difference.

## Results

There were 49 females (61%) and 31 males (39%) in the study group, while 50 females (62%) and 30 males (38%) were enrolled in the control group. The mean (standard deviation [SD]) age among patients was 44.3±16.3 (range, 12–65) years, compared with 42.1±14.4 (12–65) years in the control group. The groups were statistically similar in terms of age and gender (P>0.05).

There were no statistically significant differences between the two groups in lymphocyte, platelet, MPV, or PLR values (P>0.05). In contrast, WBC, neutrophil, and NLR results were significantly higher among RAS patients than in the control group (P<0.05). Laboratory findings for both groups are presented in Table 1.

**Table 1:** Comparison of laboratory parameters between groups

	**RAS group**	**Control group**	**P-value**
WBC (K/μL)	8.65±1.78	6.69 ±1.51	<0.05
Neutrophil (K/μL)	6.56±1.62	3.80±1.20	<0.05
Lymphocyte(K/μL)	2.41± 0.64	2.17± 0.91	>0.05
Platelet (K/μL)	262.3±43.89	253.6±67.37	>0.05
MPV	8.1 ±1.8	8.70±1.2	>0.05
NLR	3.37±1.74	1.95±1.21	< 0.05
PLR	109.1±33.1	120.7±46.02	>0.05

WBC: White blood cell; MPV: Mean platelet volume; NLR: Neutrophil-to-lymphocyte ratio; PLR: Platelet-to-lymphocyte ratio

## Discussion

The aim of this study was to investigate whether NLR, PLR, or MPV levels are elevated in patients with RAS. The key finding is that NLR values were significantly higher in patients with RAS than in the control group but that there were no significant differences between groups in lymphocyte, platelet, MPV, or PLR values. RA is an inflammatory oral mucosal disease, which is characterized by painful, multiple, recurrent, and well-demarcated small, round, or ovoid ulcers. Although many theories have been proposed to explain the cause of RAS, it still remains obscure. However, immunological mechanisms are considered to play a major role in the pathogenesis of RAS (1,2). WBC and its subtypes were found to be inflammatory markers in various inflammatory diseases. Various studies in the literature have demonstrated that NLR is a new biomarker that indicates the presence of inflammation (5, 7, 10–12). Ozler et al. (12) found a significantly higher NLR value in patients with RAS compared with healthy subjects. This was similar to our study, in which NLR was significantly higher in patients with RAS compared with controls (P<0.05).

It has been suggested that platelets play an important role in the inflammatory processes. MPV is a marker of platelet count and platelet activity, and has been widely used as a new marker of inflammation (8,9). Ekiz et al. showed that patients with RAS had significantly higher MPV and erythrocyte sedimentation rate levels compared with controls (9). However, MPV findings were not statistically higher in our study group. Based on a review of the literature and to the best of our knowledge, other than the study of Ekiz et al., no other such study has been conducted to investigate the relationship between the MPV and RAS. We believe that future studies would be helpful in determining the clinical usefulness of MPV values in RAS.

PLR is also novel biomarker that can demonstrate the presence and severity of inflammation (13). To our knowledge, this is the first study to investigate the association between PLR and RAS. However, the data obtained from this study showed no significant difference in terms of PLR (P=0.512). The main limitation of this study is the inability to perform an analysis of the prognostic value of NLR, PLR, and MPV in RAS, since the study was retrospective.

## Conclusion

The present study revealed an increased NLR in a comparison of CBC findings between groups. Higher NLR values in patients with RAS suggests that this disease has an underlying inflammatory process. We believe that this parameter could be used as a cheap and simple marker of inflammation.
